# Activation of Duck RIG-I by TRIM25 Is Independent of Anchored Ubiquitin

**DOI:** 10.1371/journal.pone.0086968

**Published:** 2014-01-23

**Authors:** Domingo Miranzo-Navarro, Katharine E. Magor

**Affiliations:** Department of Biological Sciences and the Li Ka Shing Institute of Virology, University of Alberta, Edmonton, Alberta, Canada; INRA, France

## Abstract

Retinoic acid inducible gene I (RIG-I) is a viral RNA sensor crucial in defense against several viruses including measles, influenza A and hepatitis C. RIG-I activates type-I interferon signalling through the adaptor for mitochondrial antiviral signaling (MAVS). The E3 ubiquitin ligase, tripartite motif containing protein 25 (TRIM25), activates human RIG-I through generation of anchored K63-linked polyubiquitin chains attached to lysine 172, or alternatively, through the generation of unanchored K63-linked polyubiquitin chains that interact non-covalently with RIG-I CARD domains. Previously, we identified RIG-I of ducks, of interest because ducks are the host and natural reservoir of influenza viruses, and showed it initiates innate immune signaling leading to production of interferon-beta (IFN-β). We noted that K172 is not conserved in RIG-I of ducks and other avian species, or mouse. Because K172 is important for both mechanisms of activation of human RIG-I, we investigated whether duck RIG-I was activated by TRIM25, and if other residues were the sites for attachment of ubiquitin. Here we show duck RIG-I CARD domains are ubiquitinated for activation, and ubiquitination depends on interaction with TRIM25, as a splice variant that cannot interact with TRIM25 is not ubiquitinated, and cannot be activated. We expressed GST-fusion proteins of duck CARD domains and characterized TRIM25 modifications of CARD domains by mass spectrometry. We identified two sites that are ubiquitinated in duck CARD domains, K167 and K193, and detected K63 linked polyubiquitin chains. Site directed mutagenesis of each site alone, does not alter the ubiquitination profile of the duck CARD domains. However, mutation of both sites resulted in loss of all attached ubiquitin and polyubiquitin chains. Remarkably, the double mutant duck RIG-I CARD still interacts with TRIM25, and can still be activated. Our results demonstrate that anchored ubiquitin chains are not necessary for TRIM25 activation of duck RIG-I.

## Introduction

RIG-I is an intracellular detector of 5′ triphosphate RNA that activates a signaling pathway leading to the production of type I interferon and initiation of the antiviral state [1]. The three dimensional structures of RIG-I from several species [2], [3], [4], [5] provide a molecular model for RIG-I activation [6]. The pathway starts upon sensing of viral RNA by the RIG-I helicase and regulatory domains, which undergo a conformational change, acting as a molecular camshaft that uses energy from ATP hydrolysis to expose the two caspase activator recruitment domains (CARDs) to the cytoplasm [7]. Activated CARD domains of RIG-I interact with CARD domains of MAVS (VISA, CARDIF, IPS-I) [8], [9], [10], which aggregate in a prion-like structure [11]. This conformational change allows MAVS to serve as a platform to recruit the other components of the pathway in a multiprotein signaling complex [12]. Downstream, activation of IRF3 and NF-κβ transcription factors induce type I interferon and production of proinflamatory cytokines [13].

RIG-I is normally found in an auto-repressed state in a closed conformation [14] through constitutive phosphorylation by PKC-α and PKC-β kinases [15]. Upon binding of RNA and subsequent conformational change, RIG-I is dephosphorylated by PP1 phosphatase [16], triggering the activation process. TRIM25, an E3 ubiquitin ligase is critically important in RIG-I activation [17]. The carboxy terminal SPRY domain of TRIM25 interacts with the first CARD of RIG-I involving T55, and attaches K63-linked ubiquitin chains to K172 within the second RIG-I CARD domain [17]. More recently, the need for attached ubiquitin has been disputed, as human CARD domains can be activated *in vitro* through interaction with unanchored K63-linked polyubiquitin chains produced by TRIM25, a process that also requires K172 [18], [19]. RIPLET, another E3 ubiquitin ligase, is also involved in RIG-I activation through ubiquitination of RIG-I CARD domains [20], [21]. The importance of RIG-I ubiquitination is underscored by the fact that influenza nonstructural protein 1 (NS1) blocks TRIM25 and RIPLET in a species-specific manner [22], [23].

We are investigating RIG-I function and regulation in ducks, the natural host of influenza viruses, with the aim to examine influenza interference in this pathway. Waterfowl are the natural reservoir of most influenza A strains [24], [25], and have a central role in the ecology and evolution of influenza A viruses. Some highly pathogenic avian influenza strains can replicate in ducks not showing disease signs, making waterfowl the “Trojan horses” of influenza infection [26]. Highly pathogenic avian influenza strains can be lethal in chickens, and are of concern because they occasionally infect humans. In the last decade H5N1 strains have infected 641 individuals with a mortality rate close to 60% [27]. We discovered a critical difference in pathogen detection between ducks and chickens, giving a plausible explanation for their difference in susceptibility. RIG-I of ducks is functional and highly expressed upon influenza infection, while chickens apparently lack RIG-I [28]. RIG-I initiates a robust and immediate induction of IFN stimulated genes in ducks within the first 24 hours after infection [29], contributing to duck survival.

Phosphorylation–dephosphorylation [16], [30] and ubiquitination [17] are key processes in regulation of human RIG-I. The three-dimensional structure has been determined for duck RIG-I [31] facilitating structure-function comparison with human RIG-I, but the modifications required for regulation have not been examined. From an alignment of duck RIG-I with other sequences, the phosphorylation sites for inactivation are conserved (S8 and S168, corresponding to human S8 and T170, respectively) and thus we anticipate regulation by phosphorylation is conserved. However, we noted that K172, the site of activating ubquitination, was not conserved in RIG-I of ducks, zebrafinch and other species, including mouse. Thus, we wondered whether duck RIG-I was modified by TRIM25 ubiquitination, and whether ubiquitin was attached at residues other than K172. Chickens have TRIM25, but RIPLET appears to be missing from the genome [23], [32]. Here we demonstrate TRIM25 interaction is essential for activation of duck RIG-I expressed in chicken cells. However, while TRIM25 mediated ubiquitination occurs at alternate residues, the activation of RIG-I is independent of anchored ubiquitination, suggesting duck TRIM25 is involved in production of unanchored K63-linked ubiquitin chains.

## Results

### Duck RIG-I(N) Induces Interferon-beta in Chicken DF1 Cells

**Figure 1:**
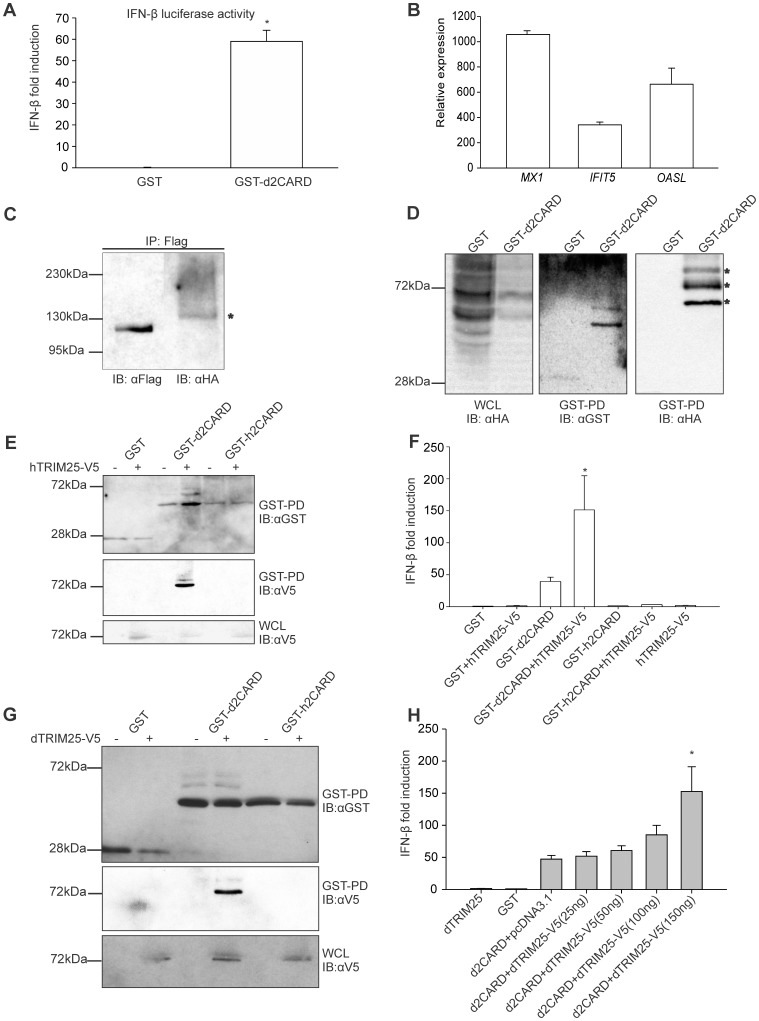
RIG-I 2CARD induces innate immune genes and ubiquitination by TRIM25 increases activation. A. Chicken IFN-β promoter activity in DF-1 cells transfected with GST or GST-d2CARD, shown as mean fold induction (±SD) from three independent experiments (n = 3) (* indicates P<0.001). B. Expression of innate immune genes (*MX1*, *IFIT5*, and *OASL*) upon signaling by duck 2CARD is shown relative to cells transfected with GST alone. Results are representative of three independent experiments and error bars show RQ_min/max_ at a 95% confidence level. C. Extracts of DF1 chicken cells transfected with duck FLAG-RIG-I together with HA-ubiquitin were used for immunoprecipitation of RIG-I with anti-FLAG and immunoblotting with anti-FLAG and anti-HA. Asterisk indicates ubiquitinated RIG-I. D. GST or GST-d2CARD and HA-Ubiquitin were transfected into DF-1 cells and used for GST pulldown and immunoblotting. GST-d2CARD is ubiquitinated as indicated by presence of larger bands in anti-GST or anti-HA blots (indicated with *). E. Chicken DF1 cells transfected with GST or GST 2CARD fusion constructs from duck (d2CARD) or human (h2CARD) and V5-epitope tagged human TRIM25 were used for GST pulldown and immunoblotting with anti-GST and anti-V5 antibodies. Duck 2CARD is ubiquitinated and associates with human TRIM25. F. Chicken IFN-β promoter activity in DF-1 cells transfected with GST-d2CARD or GST-h2CARD and human TRIM25-V5, show GST-d2CARD is further activated by the presence of hTRIM25-V5 (* indicates P<0.001). Human 2CARD is less active and not ubiquitinated in chicken cells. Data are the mean ± SD (n = 3). G. GST pulldown and immunoblot demonstrating interaction of duck TRIM25-V5 with duck CARD domains but not with human CARD domains. H. Duck TRIM25 significantly activates d2CARD compared to d2CARD alone (* indicates P<0.001). Luciferase assay was performed using the chIFN-β promoter luciferase reporter and increasing amounts of duck TRIM25-V5 plasmid (between 25 ng and 150 ng) with a fixed amount of GST-d2CARD plasmid (5 ng). All d2CARD transfections show a statistically significant activation of the chIFN-β promoter compared to the GST control (P<0.005). Data are the mean ± SD (n = 3).

Chickens lack the intracellular RNA detector RIG-I, which is present in ducks [28], but have MDA5 [33], which converges on the same signalling pathway downstream of MAVS. We previously reported the reconstitution of the pathway, and induction of an innate immune response in chicken DF1 cells transfected with duck RIG-I and RIG-I ligand [28], [34]. The N-terminal part of human RIG-I, RIG-I(N), is constitutively active and induces an innate immune response in the absence of the helicase and regulatory domains of RIG-I [17]. Exploring the possibility of similar behavior of duck RIG-I(N), we generated a GST-d2CARD construct and transfected it into the chicken embryonic fibroblast cell line, DF1. Using the dual luciferase assay with a chicken IFN-β promoter in a luciferase reporter plasmid (pGL3-chIFN-β) [28] we detected increased chicken IFN-β promoter activity in the cells transfected with the GST-d2CARD compared to the GST plasmid (Fig. 1A). We compared the expression of three interferon-stimulated-genes (ISG) in cells expressing GST-d2CARD to those with GST alone, and detected vast upregulation of *MX1, IFIT5* and *OASL* (Fig. 1B), suggesting that duck CARD domains constitutively activate the RIG-I pathway.

### Duck RIG-I is Ubiquitinated in Chicken DF1 Cells

Activation of human RIG-I involves polyubiquitin chains attached at lysine 172 of the second CARD domain [17], but this residue is absent in duck RIG-I. To determine whether duck RIG-I is modified by attached ubiquitin, we examined ubiquitination of full-length duck Flag-RIG-I using HA-tagged ubiquitin (HA-Ub) and immunoprecipitation with anti-Flag antibodies. We co-transfected DF1 cells with pcDNA3.1 Hygro+ Flag-RIG-I and pcDNA HA-Ub [35] and used cell extracts for immunoprecipitation and western blotting. We detected ubiquitination as a smear of larger forms of RIG-I in the anti-HA immunoblot (Fig. 1C). To determine whether ubiquitination within the CARD domains was involved in regulation, we performed GST-pulldown from extracts of cells transfected with GST-d2CARD and GST control plasmids. We detected ubiquitinated bands in the anti-GST and anti-HA western blots (Fig. 1D). These results demonstrate that duck RIG-I CARD domains lacking the Lys 172 residue are nonetheless ubiquitinated in chicken cells under conditions where the RIG-I pathway is active.

### Human TRIM25 Increases Ubiquitination of Duck CARD Domains

TRIM25, an E3 ubiquitin ligase, is involved in activation of human RIG-I through attachment of polyubiquitin chains [17]. To determine whether duck RIG-I is modified by human TRIM25 in chicken DF-1 cells, we used a GST-pulldown assay. We observed larger bands corresponding to ubiquitinated duck CARD domains on the immunoblot, when DF1 cells were co-transfected with human TRIM25-V5 and GST-d2CARD plasmids (Fig. 1E). To investigate whether duck RIG-I and human TRIM25 interact, we immunoblotted for the V5 epitope on TRIM25, and the pulldown of human TRIM25-V5 with duck CARD domains indicated a strong interaction between these proteins. Surprisingly, we detected no larger ubiquitinated forms, nor interaction between human CARD domains and human TRIM25 in extracts of chicken DF1 cells transfected with GST-h2CARD, suggesting that an additional factor needed for human TRIM25 and h2CARD interaction is not conserved in chicken cells (Fig. 1E). We confirmed that h2CARD was ubiquitinated and interacts with human TRIM25 in HEK293 cells demonstrating these constructs were functional (data not shown). We observed increased activation of the chicken IFN-β promoter upon co-transfection of human TRIM25-V5 and GST-d2CARD, and no chicken IFN-β promoter induction with h2CARD and hTRIM25-V5 (Fig. 1F). These results together suggest that duck CARD domains are activated by TRIM25 activity through modification involving ubiquitin chains.

### Duck TRIM25 Interacts with and Increases Activation of Duck CARD Domains

Our results above suggest that chicken and human TRIM25 can function in the activation of duck RIG-I, however, the function of duck TRIM25 is unknown. We cloned duck TRIM25 into an expression vector with a C-terminal V5 tag to investigate its interaction with duck RIG-I. GST-pulldown clearly showed that duck TRIM25 interacts with duck RIG-I CARD domains but not with human CARD domains (Fig. 1G). We observed increased RIG-I activity in a dose dependent manner, with transfection of increasing amounts, up to 150 ng of dTRIM25 plasmid, indicated by induction of the chicken IFN-β promoter (Fig. 1H). Higher amounts of duck TRIM25 (500 ng) abrogated the induction of the RIG-I pathway (data not shown), as previously described for human TRIM25 [36]. These results demonstrate that duck TRIM25 has a role in the activation of the RIG-I pathway.

### A Splice Variant of Duck RIG-I Cannot Interact with TRIM25

**Figure 2:**
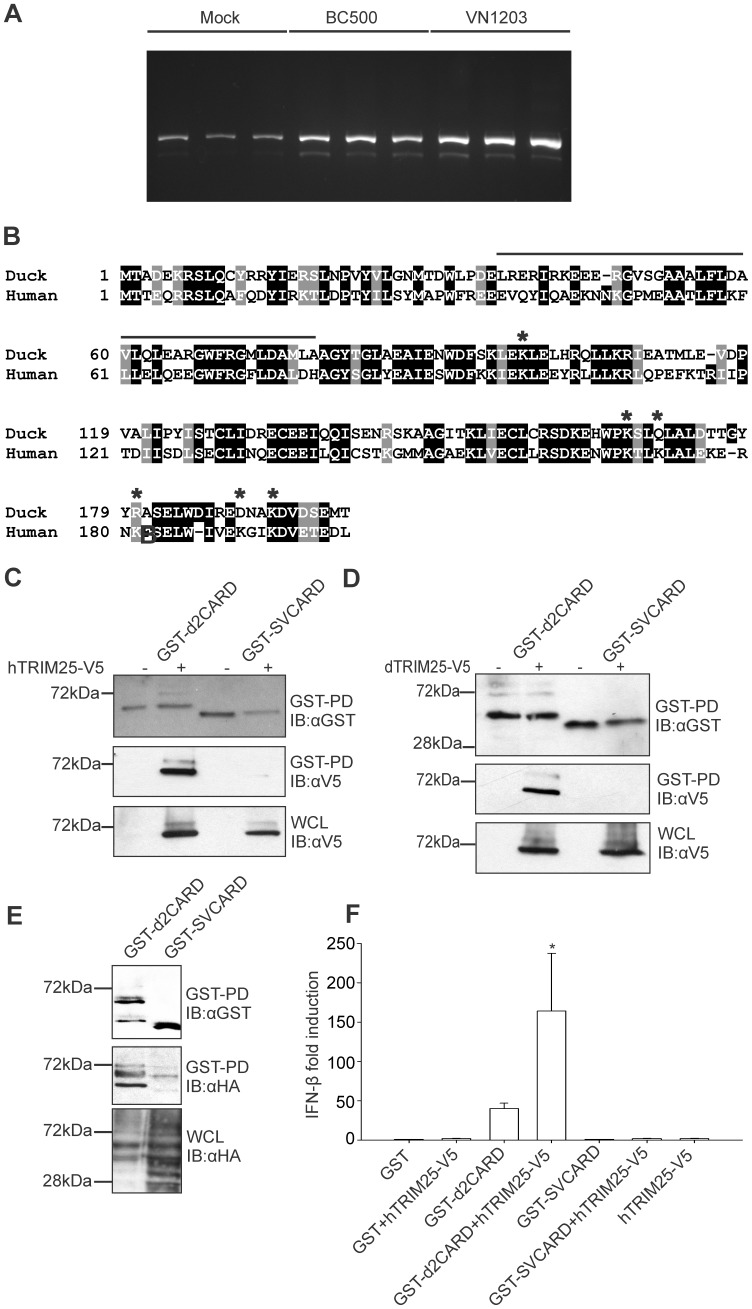
RIG-I splicing variant is not ubiquitinated and cannot activate innate immune signaling. A. Reverse transcription PCR showing amplification across exon 2 of duck RIG-I in lung tissue of ducks that were mock challenged, or infected with influenza A virus strains, BC500 or VN1203. B. Alignment of sequences of duck and human RIG-I CARD domains. Exon 2 sequence missing in splicing variant is overlined and Lys residues ubiquitinated in human RIG-I are marked with an asterisk. C. GST pulldown followed by immunoblotting indicates that GST-d2CARD interacts with human TRIM25-V5, while this interaction is greatly reduced for the splicing variant SVCARD. D. GST pulldown and immunoblotting showing no interaction between duck RIG-I CARD splice variant (SVCARD) and duck TRIM25-V5. E. GST-d2CARD is ubiquitinated while splicing variant is not. F. GST-2CARD activates the chIFN-β promoter in DF-1 cells, while splicing variant does not. Human TRIM25 significantly increased the chIFN-β promoter activity of d2CARD, but not SVCARD, compared to d2CARD alone (* indicates P<0.001). Data are the mean ± SD (n = 3).

A splice variant deleting exon 2, the region necessary for interaction with TRIM25 including the residue T55 required for interaction and ubiquitination of TRIM25, was identified in human RIG-I [37]. The production of the splice variant, which is a dominant inhibitor of the RIG-I mediated antiviral IFN response, was proposed to be a mechanism of down regulation of RIG-I activity [37]. We previously noted that ducks have alanine instead of threonine at position 55 [28], which functions suboptimally in the human RIG-I T55A mutant [37]. To determine whether a similar splice variant exists in ducks and whether it forms a non-functional RIG-I, we examined transcripts present in tissues from influenza-infected ducks from a previous experiment [28]. RNA from lung tissues taken at 3 dpi was obtained from three groups of 3 ducks -mock infected with PBS, infected with low pathogenic avian influenza A/British Columbia 500/2005 (H5N2) (BC500), or highly pathogenic avian influenza A/Vietnam 1203/2004 (H5N1) (VN1203), and reverse-transcription PCR was used to amplify across RIG-I. We observed accumulation of the splice variant in tissues from ducks infected with both viruses (Fig. 2A). The duck splice variant was confirmed by sequencing to be equivalent to its human counterpart, missing a portion of the first CARD domain (exon 2) that is essential for TRIM25 interaction [37](Fig. 2B).

### Duck RIG-I Splice Variant Cannot Activate Innate Immune Signaling

The human RIG-I splice variant does not interact with TRIM25 and is not ubiquitinated; therefore we examined the duck splice variant by GST-pulldown and immunoblotting. We observed a decrease in the interaction between the duck splice variant CARD domains (SVCARD) and human TRIM25 (Fig. 2C) or duck TRIM25 (Fig. 2D), compared with the intact GST-d2CARD fusion protein. We examined the attachment of HA-tagged ubiquitin to the SVCARD, in transfection followed by anti-HA immunoblotting. The ubiquitination of the splice variant was severely decreased in transfected DF1 cells (Fig. 2E). The activation of chIFN-β promoter for the intact CARD domains and the splice variant, relative to GST alone were compared. We observed total abrogation chIFN-β activation for the splice variant, even with addition of human TRIM25 (Fig. 2F). These results demonstrate that ducks have a splice variant of RIG-I expressed after infection. The abrogation of the innate immune response by the splice variant supports the idea that duck TRIM25 and RIG-I interact similarly to those of humans, and suggests the splice variant accumulates to down regulate the innate immune response. We explored this possibility by cotransfecting GST-d2CARD with increasing amounts of SVCARD The SVCARD does not bind to TRIM25, thus does not compete with the interferon response produced by GST-d2CARD (Fig. S1A.). We expected that the full-length SVRIG-I would bind ligand, but being unable to signal, would dampen the interferon response, as the human SVRIG-I does in competition with native RIG-I in HEK293T cells infected with Sendai virus [37]. Because duck cell lines are not available, we transfected Flag-RIG-I and increasing amounts of the splice variant RIG-I in chicken DF-1 cells, and stimulated with RIG-I ligand or control RNA. We were unable to demonstrate significant interference in RIG-I signaling by the RIG-I splice variant (Fig. S1B).

### Duck RIG-I is Ubiquitinated at Lysine 167 and Lysine 193

**Figure 3:**
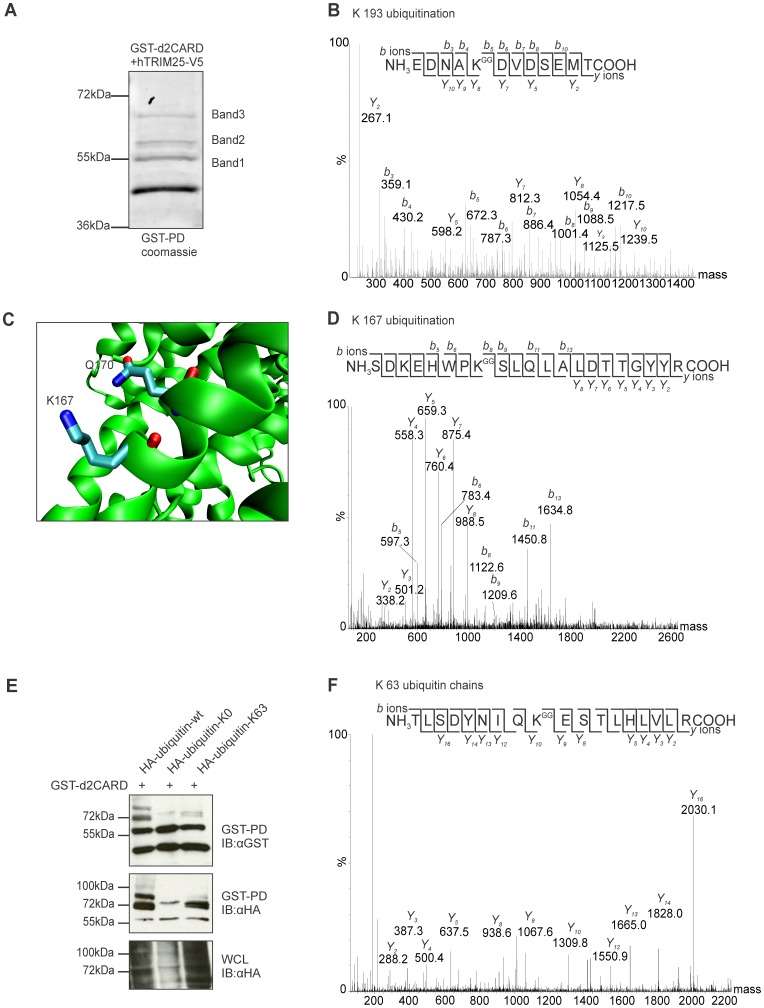
Duck RIG-I CARD domains are ubiquitinated at K167 and K193. A. Coomassie stained gel showing the different ubiquitinated forms of the duck RIG-I CARD domains. B. MaxEnt3 deconvoluted MS/MS spectra of a peptide bearing the typical diglycine signal for ubiquitination at Lys 193, showing the singly-charged forms of the B and Y ions detected. C. Three-dimensional detail of duck RIG-I structure in the region of Q170 (red), corresponding to human K172, and the closest lysine, K167 (blue) (PDB code:4a2w) [31]. Figure was created using Protean 3D from DNA Lasergene 9. D. MaxEnt3 deconvoluted MS/MS spectra of a peptide showing the ubiquitination signal for K167. E. GST pulldown of duck CARD domains from cells co-transfected with HA-ubiquitin, or HA-ubiquitin-K0 (all lysines mutated) or HA-ubiquitin K63 (only lysine 63 intact). Band 2 in the GST pulldown is a doublet and the lower band is missing in the absence of K63-linked ubiquitin chains. F. MaxEnt3 deconvoluted MS/MS spectra of a peptide of ubiquitin recovered from band 2 showing the diglycine signal at Lys 63, indicating the presence of Lys 63-linked polyubiquitin.

To determine the location of attached ubiquitin in duck CARD domains, the ubiquitinated forms of GST-d2CARD modified by human TRIM25-V5 were purified and analyzed by mass spectrometry. Three bands, larger than the GST-d2CARD protein, were detected in a coomassie stained polyacrylamide gel (Fig. 3A). These bands were equivalent in size to the ubiquitinated bands detected by western blot. We detected ubiquitin and GST-d2CARD fusion protein in all three bands, confirming ubiquitination of the duck CARD domains. Within the human CARD domains six lysines can be ubiquitinated (K99, K169, K172, K181, K190, and K193) in cells with active TRIM25 [37]. In duck CARD domains, only three lysines are conserved, K98, K167 and K193, equivalent to human K99, K169 and K193, respectively (Fig. 2B). In all three bands, we recovered Lys 193 with a diglycine modification tag indicating anchored ubiquitination (Fig. 3B). Lys 167 is the Lys nearest to the missing Lys 172, and its localization within the three-dimensional structure of duck RIG-I CARD domains [31] is close to Lys 172 (Fig. 3C). Lys 167 was ubiquitinated in band 1 (Fig. 3D). K98 was not ubiquitinated in any band. Also no ubiquitinated forms of K154 and K164, important for RIG-I activation by RIPLET [38], were present in any of the analyzed samples. These results demonstrate that duck CARD domains are ubiquitinated at Lys 167 and Lys 193 under activation conditions, and these Lys appear to be the only anchored ubiquitination sites within the CARD domains with a potential role in duck RIG-I activation.

### K63-linked Polyubiquitin Chains were Isolated with Duck RIG-I CARD

Human RIG-I is activated through the attachment of anchored K63-linked polyubiquitin chains to the second CARD domain at K172 [17]. To examine the nature of the ubiquitin bound to RIG-I CARD domains, we co-transfected the GST-d2CARD and HA-ubiquitin wildtype and mutants [39]. The GST pulldown using HA-ubiquitin-WT shows the same ubiquitinated bands detected in the coomasie stained gel above. The HA-ubiquitin-K0 mutant, with all lysines mutated, had 2 bands in the HA blot of the pulldown that likely correspond to monoubiquitin attached at K167, at K193 or at both lysines (larger band) (Fig. 3E). The HA-ubiquitin-K63 mutant, with only the K63 lysine intact allowing formation of K63-linked polyubiquitin, has two additional bands, the lower part of the doublet in band 2 and band 3. Analyzing the MS/MS results we detect a strong signal for K63-linked polyubiquitin chains in band 2 (Fig. 3F). The size of the lower part of band 2 is consistent with the linkage of two ubiquitin molecules in a polyubiquitin chain.

### Duck RIG-I CARD K167R/K193R Double Mutant is not Ubiquitinated

**Figure 4:**
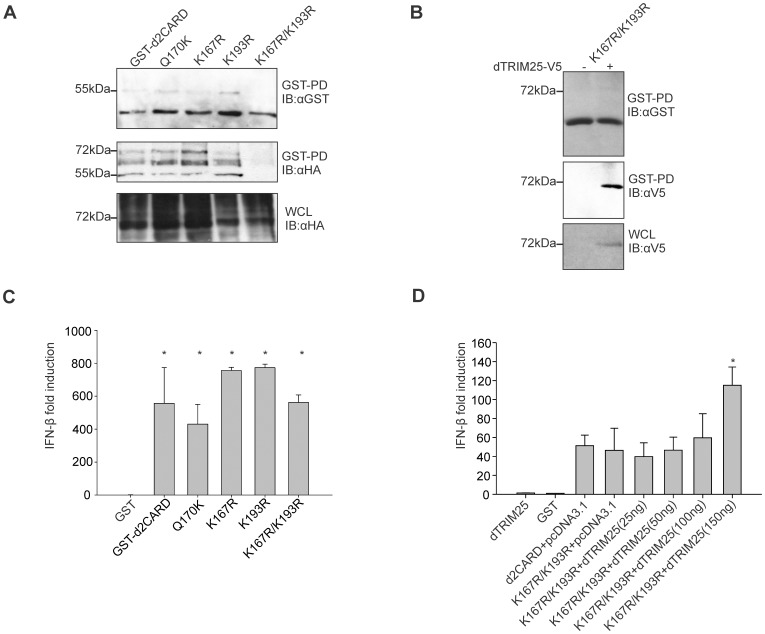
Ubiquitination but not activity of duck 2CARD is lost in double K-R mutant. A. GST pulldown and immunoblotting with anti-GST and anti-HA of samples from cells transfected with different GST-d2CARD mutants (Q170K, K167R, K193R, K167R/K193R) and HA-Ub plasmids showing a loss of ubiquitination only in the double mutant. B. GST pulldown and immunoblotting of K167/K193R mutant showing interaction with duck TRIM25-V5. C. GST-d2CARD and mutants Q170K, K167R, K193R, K167/193R activate the chIFN-β promoter when co-transfected into chicken DF-1 cells. Data represent the mean ± SD (n = 3). All mutants tested significantly activate the chIFN-β promoter compared with the GST control (P<0.05). None of the CARD mutants are statistically different compared to d2CARD. D. Luciferase assay was performed using the chIFN-β promoter and increasing amounts of duck TRIM25-V5 plasmid (between 25 ng and 150 ng) with a fixed amount of K167R/K193R mutant CARD domain plasmid (5 ng). Results are the mean±SD (n = 3) and dTRIM25 significantly increased activation of chIFN-β compared to d2CARD (* indicates P<0.005). All d2CARD samples show a statistically significant activation of the chIFN-β promoter compared with the GST control (P<0.05).

To ascertain the importance of sites for attachment of anchored polyubiquitin chains in the activation of duck CARD domains in DF1 chicken cells, we created a set of mutants and examined their ubiquitination. We first created a mutant, Q170K, to restore the equivalent of the human RIG-I lysine 172. This mutant did not show an increased ability to activate the RIG-I pathway and ubiquitinated bands look identical to those of the wild type construct. We then mutated each of the lysines, K167, and K193 independently, and together in a double mutant. Both of the single mutants (K167R, K193R) showed identical patterns of bands demonstrating ubiquitination, but the K167R/K193R double mutant lost all larger bands indicating it was not ubiquitinated, nor were K63 polyubiquitin chains attached (Fig. 4A). These results demonstrate that both K167 and K193 are attachment sites for bound ubiquitin and polyubiquitin chains in duck RIG-I CARD domains.

### Duck RIG-I CARD K167R/K193R Mutant can be Activated by TRIM25

To determine whether the duck CARD domains with the mutated lysines could interact with TRIM25, we cotransfected the mutant with duck TRIM25 in DF-1 cells. We confirmed the interaction of duck CARD domains bearing K167 and K193 mutations with duck TRIM25 using GST-pulldown (Fig. 4B), suggesting these lysines are not essential for duck TRIM25 interaction. No larger bands were detected in the GST-pulldown samples in the presence of duck TRIM25 indicating that duck TRIM25 does not attach ubiquitin or polyubiquitin chains at any lysine other than K167 and K193 (Fig. 4B). Exploring the importance of K167 and K193 in the activation of RIG-I, we used the dual luciferase assay and examined chIFN-β promoter induction in cells transfected with the different mutants. None of the mutants lost its ability to activate the RIG-I pathway, as indicated by 400-fold to 800-fold induction of the chicken IFN-β promoter (Fig. 4C). These results suggest that ubiquitination of K167 and K193 is not essential for activation of RIG-I CARD domains. We show the ability of duck TRIM25 to increase the activity of the double mutant K167R/K193R (Fig. 4D). Taken together these results demonstrate that duck TRIM25 is activating duck RIG-I through a mechanism that does not require anchored K63-linked polyubiquitin chains.

## Discussion

Here we examine regulation of RIG-I in the natural host of avian influenza. Human RIG-I is activated by K63-linked polyubiquitin attached at lysine 172 in the second CARD domain [17], or interaction with unanchored K63-linked polyubiquitin chains, also involving lysine 172 [19], a residue that is missing in ducks and other birds. We demonstrate that chicken and duck TRIM25 are functional, and duck CARD domains are ubiquitinated. The duck TRIM25-RIG-I interaction plays a role in the activation of IFN-β production as demonstrated by the nonfunctional splice variant of RIG-I, which is unable to interact with TRIM25, and does not induce IFN-β. Using mass spectrometry to analyze ubiquitinated CARD domains, we determined that K167 and K193 were the ubiquitination sites of duck CARD domains. We showed that mutation of each site independently did not disrupt ubiquitination, however a double mutant with both K167R and K193R, was unable to undergo ubiquitination. Surprisingly, this double mutant retained activity and the ability to be activated by duck TRIM25. Thus, duck TRIM25 can activate RIG-I through a process that does not depend on attachment of ubiquitin chains to the CARD domains of RIG-I.

Duck CARD domains undergo robust ubiquitination and activate IFN-β in DF1 chicken cells indicating that chicken cells contain all the proteins required for the activation and downstream signaling of RIG-I. Previously, Rajsbaum et al. [23] showed that knockdown of chicken TRIM25 decreased IFN production in chicken cells. Since chickens lack RIG-I, this unexpected observation highlights that MDA5 and RIG-I share the same activation pathway, and likely TRIM25 is also involved in activation of MDA5. However, Gack et al., [17] showed MDA5 CARD domains are not modified by bound ubiquitin. Recent *in vitro* studies have shown that MDA5 and RIG-I interact with unanchored K63-linked polyubiquitins chains generated by TRIM25 [19] and are activated by the same dephosphatase [16]. The demonstration that RIG-I CARD domains are ubiqutinated in chicken cells suggests that chicken TRIM25 is activating duck RIG-I in the transfected DF-1 cells, and potentially also activates chicken MDA5.

Human TRIM25 ubiquitinated duck CARD domains and augmented their activation in chicken DF-1 cells. Surprisingly, human TRIM25 does not interact, ubiquitinate, or activate human CARD domains co-transfected in chicken DF-1 cells. This implies that some factor needed for this interaction is missing or diverged in chicken cells. Potentially, a factor needed in the ubiquitination steps is not conserved. The E2 ligases Ubc13-Uev1a and UbcH5a are needed for the activation of the RIG-I signaling pathway reconstituted *in vitro* [18]. Both E2 ligases interact with TRIM25 [17], [40]. These two E2 ligases are also partners of the E3 ligase CHIP, and in this context Ubc13-Uev1a produces K63-linked unanchored polyubiquitin chains, while UbcH5a catalyzes the formation of covalently attached polyubiquitin chains [41]. Both E2 ligases are highly conserved in chickens, and the human and duck TRIM25 are interchangeable for activation of duck RIG-I. An alternative hypothesis is that human CARD domains cannot interact with chicken MAVS, and this interaction is needed prior to attached monoubiquitination. While this idea is contrary to the existing models for RIG-I activation, the divergence of chicken and human MAVS also explains the previously observed inability of chicken MDA5 to function in human cells (VERO cells) or human MDA5 to function in chicken DF-1 cells [33]. It is worth noting that ducks, and other birds, have an alanine residue at D122, and the D122A mutant of human RIG-I(N) does not activate the IFN pathway even though it interacts with polyubiquitin chains [18], presumably because it cannot engage MAVS.

The conservation of a RIG-I splice variant present in tissue samples of influenza-infected ducks provides indirect evidence of the importance of the TRIM25 RIG-I physical interaction, and indicates the same region of duck RIG-I is involved. The RIG-I SV, lacking exon 2 cannot interact with duck TRIM25. This region of human RIG-I includes the residue T55, which is critically involved in the interaction, as T55I or T55E mutants are inactive [37]. Duck RIG-I does not have the residue T55, but has the conservative substitution alanine. The human RIG-I mutant T55A still interacts with human TRIM25 [37], but is suboptimal compared to wild type. Finally, the presence of this splice variant in tissues at 3 dpi, when the response to infection is winding down, is consistent with the hypothesis that the splice variant functions as a dominant inhibitor of full length RIG-I to prevent MAVS activation, as demonstrated for the human RIG-I splice variant [37]. However, we are unable to demonstrate inhibition of duck RIG-I signaling by the mutant SVRIG-I lacking exon 2, when both are equally expressed in chicken DF-1 cells, at amounts above the natural abundance of the transcript.

Using mass spectrometry we demonstrated that TRIM25 attaches anchored ubiquitin to the duck RIG-I CARD domains at residues K167 and K193. In band 1, we saw evidence of K167 and K193 ubiquitination, and also evidence for unubiquitinated K167, consistent with monoubiquitin attached to either of these residues, as the size of this band would predict. In band 2, we saw ubiquitination at K193 only, and a strong signal for K63-linked polyubiquitin chains. Band 2 was always seen as a doublet, and the lower band disappeared when the ubiquitin lacking all lysine residues was used, precluding production of polyubiquitin chains. The size of the upper band 2 is consistent with ubiquitin attached at both K167 and K193, while the lower band is a K63-linked polyubiquitin chain attached at one residue. Finally, in the third band we detected only ubiquitin attached at K193, and the size of this band correlated with the size of a chain of three ubiquitins. The pattern of ubiquitinated bands appeared identical to the bands arising from ubiquitination of human RIG-I, despite the ubiquitination of different residues.

We created a double mutant K167R/K193R form of the RIG-I card domains that could not be ubiquitinated by human or duck TRIM25. Independent mutation of each site (K167R or K193R) did not disrupt the attachment of ubiquitin, suggesting that attachment can occur at either of the identified sites. K167 lies close to the predicted site of phosphorylation at S168 in duck (corresponds to T170 in human RIG-I). Dephosphorylation of S168 may be a necessary step for the ubiquitination at either K167 or K193, and ubiquitination may prevent re-phosphorylation. No other lysines were found modified, and it appears that no other sites can be modified, since mutation of both lysines abrogates attachment of any ubiquitin. We also saw no attachment of ubiquitin at sites typically modified by RIPLET, as expected given the lack of RIPLET in the chicken genome [23], [32].

The double mutant form of RIG-I, that cannot be ubiquitinated, indirectly demonstrates that activation of the duck CARD domains by duck or human TRIM25 does not require any anchored ubiquitin chains. TRIM25 produces unanchored ubiquitin chains that activate RIG-I *in vitro* [18], but the importance of this has not been demonstrated *in vivo*. The double mutant of duck RIG-I CARD domains, still interacts with human or duck TRIM25 and can be activated. The most likely explanation is that duck RIG-I CARD domains are associating with unanchored polyubiquitin produced by TRIM25. This is in direct contrast to the K172R mutation of human RIG-I CARD domains that abrogates ubiquitination and activation [17], including activation by interaction with unanchored polyubiquitin [18], [19], [42].

The role of anchored ubiquitin on K172 in the activation of human RIG-I has been questioned recently [18], [19] and it was suggested that only unanchored K63-linked polyubiquitin chains are important for RIG-I activation. We demonstrated that the attachment of ubiquitin at duck RIG-I K167 and K193 is not necessary for activation of the CARD domains, but we suggest that duck RIG-I is unlikely to have ubiquitination sites without function in the cell. Indeed, the function of attached ubiquitin at K172 (human), or K167 and K193 (ducks) remains elusive, but perhaps this function is required for conformational changes leading to the activation of the intact RIG-I. The ability to generate anchored ubiquitin could be evidence of the ancestral activity of TRIM25, while activation by unanchored polyubiquitin chains was later derived. In addition, TRIM25 can undergo autoubiquitinylation with polyubiquitin chains [43]. Recent enzymatic studies suggest that TRIM25 generates the ubiquitin chains anchored to the E2 ligase and then they are transferred to the substrate [44], generating a plausible scenario for the coexistence of the two types of ubiquitin chains: some of the transferred chains will be attached to RIG-I and some will remain unanchored. Another possibility is that TRIM25 produces K63-linked polyubiquitin chains attached to RIG-I and an unknown deubiquitinating enzyme produces the unanchored chains [45]. Our data, with activation of duck RIG-I CARD domains in the absence of ubiquitin attachment sites makes the latter model unlikely.

In an alignment of RIG-I CARD domain amino acid sequences (Fig. S2), it is noteworthy that the lysines equivalent to human K169 and K193 are highly conserved across species, while the lysine equivalent to human K172 is not present in birds or rodents. RIG-I from avian species, including ducks, geese and zebrafinch, all have the equivalent lysine K169 and K193, and have the motif KSLQ, in the RIG-I regulatory site equivalent to the human KT^170^LK^172^. Turtle (*Chelonia mydas*) has the conserved K169 and K193, and has the motif KTFH. Teleost species, such as zebrafish (*Danio rerio*) have the sequence KVLK, and the K193 is not conserved. Mouse and rat have K169 and the motif KVLQ. Among RIG-I sequences for all species to date, K169 is conserved in all, and K193 is missing only in wild boar and rat. Serine or threonine at the site of phosphorylation of human RIG-I (T170) is well conserved among most species, but is missing in rodents and bats. Clearly, the RIG-I regulatory motif has changed over evolutionary time, possibly due to selection pressure from viruses intent on disrupting this regulation.

To suggest a model for duck RIG-I activation consistent with our observations, dephosphorylation at serine residues S8 and S168 allows RIG-I to interact with MAVS, where it undergoes monoubiquitination of lysine residues K167 or K193 in the RIG-I CARD domain by TRIM25, and attachment or association with polyubiquitin chains. Alternatively, additional ubiquitin moieties are attached to the monoubiquitin bound at either site. The activation of duck RIG-I in the absence of bound ubiquitin, which is in contrast to human RIG-I lacking K172, may reflect different specificity for duck RIG-I in binding unanchored polyubiquitin chains for activation. Indeed, the activation of duck RIG-I in the absence of attached polyubiquitin chains may be a mechanism to evade viral subversion of ubiquitin pathways [23], such as NS1 from H5N1 avian influenza, which preferentially interacts with TRIM25 in chicken cells, and decreases IFN production [23], even in the absence of RIG-I [28].

## Materials and Methods

### Ducks and Avian Influenza Virus Infections

cDNA samples prepared from lung tissue taken from ducks infected with avian influenza A/Vietnam 1203/04 (H5N1) (VN1203) or A/British Columbia 500/05 (H5N2) (BC500) were obtained from a previous study done in collaboration with Dr. Robert G. Webster at St. Jude Children’s Research Hospital, Memphis, TN [28]. All animal experiments in that study were approved by the Animal Care and Use Committee of St. Jude Children’s Research Hospital and performed in compliance with relevant institutional policies, National Institutes of Health regulations, and the Animal Welfare Act.

### Cell Culture and Transfection

DF-1, a chicken embryonic fibroblast cell line derived from East Lansing strain eggs [46], was maintained in DMEM plus 10% FBS. Cells were seeded overnight in 24-well (2×10^5^) or 6-well (8×10^5^) plates and 24 h later cells were transfected with 1 µg/well (24-well plates) or 1.5 µg/well (6-well plates) of each of the indicated DNA constructs using Lipofectamine 2000™ reagent (Invitrogen) (ratio 1∶2.5).

### Plasmids

pcDNA3.1 (Hygro+) (Invitrogen) was the backbone plasmid used in all the constructs presented in this work and Phusion® High-Fidelity PCR Master Mix (NEB) was used in all PCR and site-directed mutagenesis reactions. To express the GST protein (pEBG plasmid) in DF1 chicken cells, a 716 bp PCR fragment containing the ORF coding for GST and the multicloning site from pEBG were amplified and cloned into pcDNA3.1 (Hygro+) using NheI-XhoI restriction sites encoded in the primers. The pcDNA3.1 (Hygro+)-GST construct was used for cloning the CARD domains (600 bp) of human RIG-I [17] and duck RIG-I [28] using BglII-ClaI restriction sites encoded in the primers to produce GST-CARD fusion constructs, GST-hCARD- and GST-d2CARD in pcDNA3.1 (Hygro+)-GST using BamHI-ClaI sites.

Duck TRIM25 was amplified and cloned into pCR 2.1-TOPO (Invitrogen) using cDNA from lung samples collected at 1 day post infection with H5N1 virus A/Vietnam/1203/04 [28]. The duck TRIM25 was cloned into pcDNA3.1 (Hygro+) using NheI-NotI containing primers with a V5 epitope coding sequence in the reverse primer.

The duck RIG-I splice variant was detected in cDNA prepared from a lung sample collected at 3 dpi with A/Vietnam/1203/04 (H5N1) [34]. PCR products across the intron were fully sequenced to confirm the same deletion of residues corresponding to exon 2 seen in human RIG-I [37]. For construction of the GST-splice variant CARD domain construct, the duck CARD domains cloned in pCR 2.1-TOPO with added sites BglII-ClaI (TOPO-d2CARD) was used as template for a PCR with primers flanking the second exon of RIG-I and facing outwards. The PCR product was treated with T4 polynucleotide kinase (NEB) to generate PCR fragments susceptible to ligation. The TOPO-SVCARD was digested with BglII-ClaI and the fragment containing SVCARD domains was cloned into pcDNA3.1 (Hygro+)-GST using BamHI-ClaI. The full length Flag-RIG-I was cloned into pcDNA3.1 (Hygro+) using NheI-NotI containing primers with a Flag epitope coding sequence in the forward primer using pcDNA-RIG-I [28] as template. For construction of the splice variant version of the full-length Flag-RIG-I, the duck full length Flag-RIG-I cloned in pCR 2.1-TOPO with added sites NheI-NotI (TOPO-Flag-RIG-I) was used as template for a PCR with primers flanking the second exon of RIG-I facing outwards. The PCR product was treated with T4 polynucleotide kinase (NEB) to generate PCR fragments susceptible to ligation. The TOPO-Flag-RIG-I was digested with NheI-NotI and the fragment containing full length Flag-SVRIG-I was cloned into pcDNA3.1 using NheI-NotI. For induction of full length Flag-RIG-I and Flag-SVRIG-I constructs, 5′ppp-dsRNA and dephosphorylated dsRNA control (InvivoGen) were used.

All CARD mutants used in this work (Q170K, K167R, K193R, K167R/K193R) were made using an adapted protocol for site-directed mutagenesis [47] using TOPO-dCARD-GST as template. Mutations were confirmed by sequencing and the mutant CARD domains were cloned into the pcDNA3.1 (Hygro+)-GST construct using ΔBamHI/BglII-ClaI sites. Constructs were confirmed by complete sequencing. Human GST-CARD and human TRIM25-V5 were kindly provided by Dr. Michaela U. Gack. pcDNA3.1-HAUB (Addgene plasmid 18712) [35] was used to detect ubiquitinated forms of RIG-I. pRK5-HA-Ubiquitin-WT (Addgene plasmid 17608), pRK5-HA-Ubiquitin-K0 (Addgene plasmid 17603), pRK5-HA-Ubiquitin-K63 (Addgene plasmid 17606) [39] were used to study the nature of the ubiquitinated forms of RIG-I.

### Immunoprecipitation, GST Pulldown, and Immunoblotting

Flag® Immunoprecipitation kit (Sigma-Aldrich) was used for immunoprecipitation experiments. GST pulldown was performed as previously described [17]. Briefly, DF1 cells transfected with DNA constructs (one confluent 6-well plate per sample) were lysed in 1200 µl of lysis buffer (50 mM TRIS pH 7.2, 150 mM NaCl, 1% [vol/vol] Triton X-100, protease inhibitor cocktail [Roche]), followed by centrifugation at 13,000 rpm for 5 min. For GST-pulldown assays, supernatants were mixed with 50 µl of glutathione Sepharose 4B resin (GE Healthcare) equilibrated with lysis buffer, and the binding reaction mix was incubated for 3 to 4 h at 4°C. The GST-pulldown was washed three times with ice-cold lysis buffer and eluted with 25 µl 4X Laemmli buffer, followed by boiling for 10 min. For immunoblotting, proteins were separated by SDS–polyacrylamide gel electrophoresis (SDS–PAGE) and transferred to a nitrocellulose membrane. Immunodetection was achieved with anti-V5 (1∶5,000) (Invitrogen), anti-Flag (1∶2,000) (Sigma), anti-HA (1∶1000) (Sigma-Aldrich) or anti-GST (1∶1000) (Dana-Farber Monoclonal Core Facility (DG-122) antibodies and proteins were visualized by chemiluminescence using the ECL kit (GE- Healthcare).

### Q PCR and Luciferase Assay

Cells (2×10^5^) were seeded overnight in 24-well plates. To quantify gene expression from transfected chicken DF1 cells, primers and probes specific for *Mx1*, *IFIT5* and *OASL* were used for Q-PCR as previously described [34]. Reverse transcription PCR for detection of RIG-I splice variant was carried out on cDNA samples previously prepared [28] from lung tissues of ducks infected with A/British Columbia 500/2005 (H5N2) and A/Vietnam 1203/2004 (H5N1), using forward (SVRIG-I.fw 5′-GAG CCT CAA CCC GGT CTA C-3′) and reverse primer (SVRIG-I.rev 5′-GGT AGC TCC GAG CCT TCT TT-3′) flanking duck RIG-I exon 2. Luciferase activity was measured using the Dual-Luciferase Reporter Assay System (Promega) 24 h after transfection with the chicken IFN-β promoter luciferase reporter plasmid (pGL3-chIFNβ) derived from the chicken *IFN2* gene as previously described [28], [33], [48]. Briefly, DF1 cells were transfected with fixed amounts of pGL3-chIFNβ (150 ng), the synthetic Renilla luciferase reporter construct phRTK as internal control (10 ng) and GST-d2CARD or K167R/K193R mutant (5 ng). Cells were also transfected with increasing amounts of duck TRIM25-V5 construct (between 25 ng and 150 ng) and variable amounts of pcDNA3.1 (Hygro+) (Invitrogen) to normalize the amount of transfected DNA.

### Mass Spectrometry

The GST resin purified protein was separated by SDS-PAGE, and the bands corresponding to the ubiquitinated forms of duck RIG-I GST-CARD domains fusion protein were in-gel digested with trypsin (Promega) using the adapted protocol of Shevchenko et al. [49], [50]. Samples were analyzed using a hybrid quadrupole orthogonal acceleration time-of-flight mass spectrometer (Waters, UK) equipped with a nanoACQUITY Ultra Performance liquid chromatography system (Waters, Milford, MA) as previously described [50]. Briefly, 2 µl of the peptide solution was injected into a VanGuard micro precolumn C18 cartridge connected to a 75 µm i.d.×150 µm Atlantis dC18 column (Waters, Milford, MA). Solvent A was 0.1% formic acid in water, and solvent B was 0.1% formic acid in acetonitrile. After 1 min trap wash in the precolumn with solvent A at flow rate of 10 µl/min, peptides were separated using solvent gradient and electrosprayed to the mass spectrometer at a flow rate of 350 nl/min. The collision energy used to perform MS/MS depended on the mass and charge state of the eluting peptides. The instrument was calibrated every 1 min with GFP and LecErK using the LockSpray. MassLynx (Waters MassLynx V4.1) was used for the data acquisition and analysis.

## Supporting Information

Figure S1 **The RIG-I splice variant is not a dominant inhibitor of RIG-I.** A. The SVCARD is not active, and is not acting as a dominant inhibitor of RIG-I. The dual luciferase assay was performed using the chIFN-β promoter and increasing amounts of SVCARD plasmid (25 ng to 150 ng) with a fixed amount of GST-d2CARD plasmid (150 ng). Data are mean ± SD (n = 5). GSTdCARD activates the chIFN-β promoter compared with the GST control (P<0.001). B. SVRIG-I does not act as a dominant inhibitor of duck RIG-I. Luciferase assay was performed using the chIFN-β promoter and increasing amounts of Flag-SVRIG-I plasmid (25 ng to 150 ng) with fixed amount Flag-SVRIG-I plasmid (150 ng). No significant decrease of the activation of the chIFN-β promoter was observed when increasing amounts of Flag-SVRIG-I plasmid were added. Data are mean ± SD (n = 3). All samples show activation of RIG-I by ligand compared to the pcDNA3.1+ control RNA sample (P<0.05).
(TIF)

Figure S2 **Alignment of RIG-I CARD domains from vertebrate species.** Amino acid alignment of selected sequences available in Genbank, including human (AAI07732), squirrel monkey (XP_003939778), horse (XP_001497895), wild boar (NP_998969), cat (XP_003995589), ferret (XP_004765417), Brandt’s bat (EPQ03535), black flying fox (AEW46678), rabbit (XP_002708086), mouse (BAC37205), rat (XP_216380, duck (ACA61272), goose (AEG75816), zebrafinch (XP_002194560), green turtle (EMP30788), zebrafish (XP_002666571). Asterisks indicate ubiquitinated residues of human RIG-I CARD domains in the presence of active TRIM25. The plus symbol indicates the D122 residue and the circumflex indicates the regulatory phosphorylation sites.
(TIF)
