# Supplementary figures and images for: Activation of Duck RIG-I by TRIM25 Is Independent of Anchored Ubiquitin
Source: PLoS One. 2014 Jan 23;9(1):e86968. doi: 10.1371/journal.pone.0086968 (PMC3900705; doi:10.1371/journal.pone.0086968)

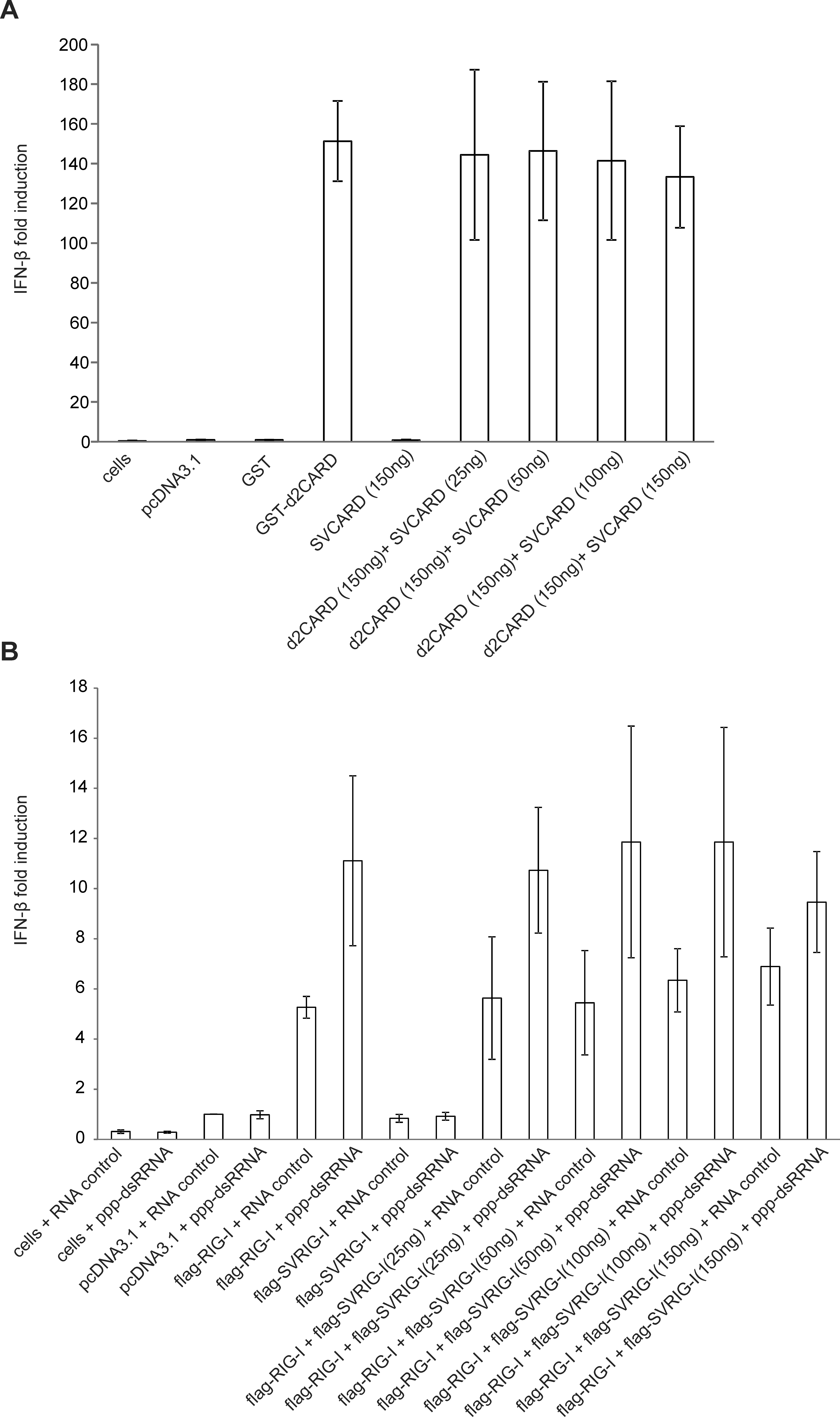

Supplement: Figure S1 — The RIG-I splice variant is not a dominant inhibitor of RIG-I. A. The SVCARD is not active, and is not acting as a dominant inhibitor of RIG-I. The dual luciferase assay was performed using the chIFN-β promoter and increasing amounts of SVCARD plasmid (25 ng to 150 ng) with a fixed amount of GST-d2CARD plasmid (150 ng). Data are mean ± SD (n = 5). GSTdCARD activates the chIFN-β promoter compared with the GST control (P<0.001). B. SVRIG-I does not act as a dominant inhibitor of duck RIG-I. Luciferase assay was performed using the chIFN-β promoter and increasing amounts of Flag-SVRIG-I plasmid (25 ng to 150 ng) with fixed amount Flag-SVRIG-I plasmid (150 ng). No significant decrease of the activation of the chIFN-β promoter was observed when increasing amounts of Flag-SVRIG-I plasmid were added. Data are mean ± SD (n = 3). All samples show activation of RIG-I by ligand compared to the pcDNA3.1+ control RNA sample (P<0.05). (TIF) [file pone.0086968.s001.tif]

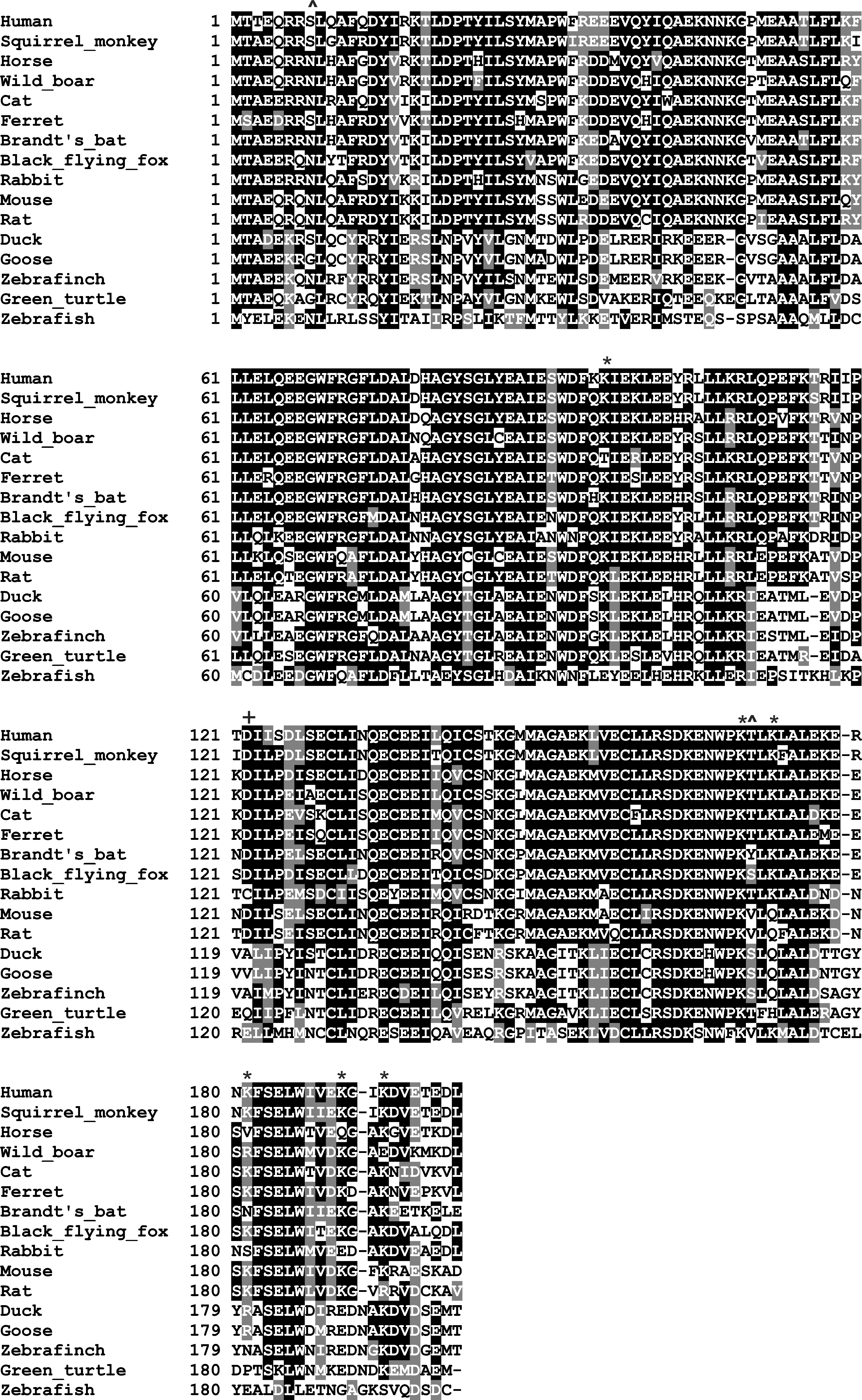

Supplement: Figure S2 — Alignment of RIG-I CARD domains from vertebrate species. Amino acid alignment of selected sequences available in Genbank, including human (AAI07732), squirrel monkey (XP_003939778), horse (XP_001497895), wild boar (NP_998969), cat (XP_003995589), ferret (XP_004765417), Brandt’s bat (EPQ03535), black flying fox (AEW46678), rabbit (XP_002708086), mouse (BAC37205), rat (XP_216380, duck (ACA61272), goose (AEG75816), zebrafinch (XP_002194560), green turtle (EMP30788), zebrafish (XP_002666571). Asterisks indicate ubiquitinated residues of human RIG-I CARD domains in the presence of active TRIM25. The plus symbol indicates the D122 residue and the circumflex indicates the regulatory phosphorylation sites. (TIF) [file pone.0086968.s002.tif]
